# Community-Based Interventions to Support HIV and AIDS Orphans and Vulnerable Children (OVC) in Africa: A Systematic Review

**DOI:** 10.5334/ijic.8920

**Published:** 2025-04-29

**Authors:** Livhuwani Precious Matshepete, Lufuno Makhado, Ntsieni Stella Mashau

**Affiliations:** 1 Department of Public Health, Faculty of Health Sciences, University of Venda, 1 University Road, Thohoyandou, ZA

**Keywords:** community-based intervention, support, HIV and AIDS, orphans and vulnerable children, Africa

## Abstract

**Introduction:**

Community-based interventions to support orphans and vulnerable children were the basis for this systematic review, which was undertaken using the available literature based on different studies that outlined community-based interventions.

**Methodology:**

A systematic literature searches for quantitative, mixed methods, and qualitative studies describing the community-based intervention to support HIV and AIDS orphans and vulnerable children in Africa was performed in the following databases: Google Scholar, PubMed, EBSCOhost and Science Direct. The search utilized keywords, Boolean operators, and search engine filters, guided by inclusion and exclusion criteria as part of the search strategy. The keywords used included: community-based interventions, support (psychosocial, economic, material, and social), OVC, and Africa. A thematic approach was used to analyze common emerging themes in the community-based interventions to support HIV and AIDS orphans and vulnerable children in Africa.

**Results:**

In total, 16 studies met the inclusion criteria, documenting a range of community-based interventions to support OVC in Africa. These included food and/or food parcels, child protection, healthcare services, as well as educational, legal and psychosocial support.

**Conclusion:**

Community-based intervention support plays a crucial role in enhancing the lives of orphans and vulnerable children in Africa. Community-based intervention support needs to be integrated into a wider social service system. Further research focusing on the cost of community-based intervention support and successful programs for mainstreaming community-based intervention into a wider social system is needed.

## Introduction

In Africa, there has been an increase in the number of orphans and children who are vulnerable since the advent of Human Immunodeficiency Virus (HIV) and Acquired Immune Deficiency Syndrome (AIDS). An estimated 163 million children are living as orphans, with 17.5 million having lost one or both parents due to the HIV and AIDS pandemic [1]. This crisis requires establishing programs to support and mentor orphans and vulnerable children (OVC). However, the psychosocial needs of OVC are neglected or overlooked by the service providers [2]. In response to this crisis, there have been concerted efforts in the form of community-based interventions to mitigate the impact of HIV and AIDS on children [3]. Community-based interventions (CBOs) have therefore been identified as a strategic point for psychosocial support intervention [4,5].

Community-based interventions of OVC can take many forms, such as financial help, home care, defense against the law, dietary needs, and emotional support. Numerous studies on community-based intervention support for OVC have been conducted across Africa, including in South Africa (n = 7), Kenya (n = 1), Zimbabwe (n = 4), Nigeria (n = 2), and Uganda (n = 1) [2,6]. Notwithstanding various community-based interventions to support the OVC, the lack of support focusing on the psychosocial well-being of the OVC is still a significant concern.

OVC face a range of social and economic challenges, as well as mental anguish, stigma mourning, discrimination, loneliness, loss of educational opportunities, engagement in risky sexual behaviours, engagement in substance abuse, onerous family obligations and worry for the future, exposure to abuse and exploitations [7,8,9,10]. Communities in Africa have addressed the challenges faced by OVC by establishing support programs through community-based organisations [4,5].

Currently, orphan and vulnerable children programs have primarily focused on meeting material needs, often at the expense of addressing more delicate emotional needs such as love, attention, parental guidance, and opportunities to build self-confidence, self-esteem, hope, and self-actualisation [6]. OVC face multiple health, education and psychosocial well-being risks [3,9]. The researchers thought it was vital to focus on this topic as it addresses on issues of orphans and vulnerable children’s psychological needs. Nearly 10% of HIV-positive persons worldwide are under 18, and 17.8 million children are orphaned due to the virus [5]. A high percentage of OVC live with just one parent, ageing grandparents who require care and assistance, or any other family member who will be struggling to have basic needs [6]. The systematic review aimed to identify the community-based intervention supporting HIV and AIDS-affected OVC in Africa. The specific objective was to review the available literature to identify the types of community-based interventions supporting OVC in Africa.

Community-based interventions supporting HIV and AIDS-affected OVC demonstrate that these interventions can be systematically integrated, emphasizing the importance of community partnerships at various levels for the effective delivery of a holistic approach to supporting HIV and AIDS-affected OVC.

## Material and methods

### Information sources and search strategy

Guidelines for systematic reviews and meta-analyses were adhered to in conducting this review [11]. The search was conducted on four databases: Google Scholar, PubMed, EBSCOhost and Science Direct. The search utilized keywords, Boolean operators, and search engine filters, guided by inclusion and exclusion criteria as part of the search strategy. The keywords used were community-based interventions, support, HIV and AIDS, Orphans and Vulnerable Children and Africa.

### Eligibility criteria

The eligibility requirements were specifically based on the type of study and participant categories:

- Type of Study: The review focused exclusively on research evaluating community-based interventions supporting HIV and AIDS-affected OVC in Africa. Only publications in English, published between 2010 and 2021 (a 10-year period), were included. The study incorporated qualitative, quantitative, and mixed-methods research studies.
- Types of participants: Participants were orphans and vulnerable children in Africa who were receiving community-based interventions.

### Exclusion criteria

The systematic review excluded grey literature, studies published in languages other than English, studies conducted before 2010 or after 2021, research involving participants other than OVC, studies conducted outside Africa, and those not focusing on community-based interventions.

### Study selection

**Figure 1:**
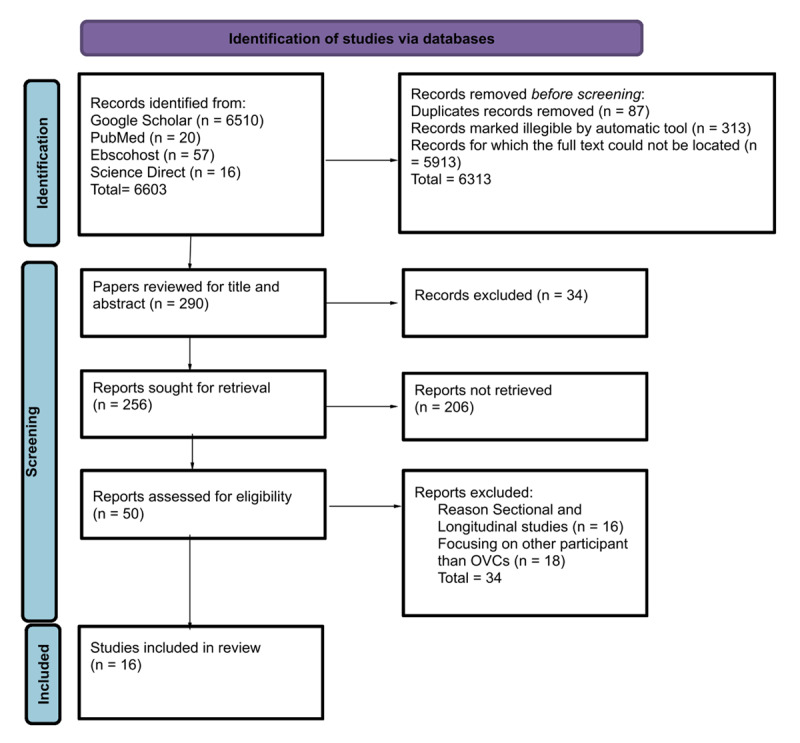
Prisma flow chart.

The study selection followed the PRISMA flow to ensure methodological rigour and quality [12]. The researchers identified records from the following databases: Google Scholar (n = 6510), PubMed (n = 20), EBSCOhost (57) and Science Direct (n = 16). The abstracts of the online studies were screened, reviewed, and examined. The researchers removed 6313 articles before the screening, 87 duplicates were removed, 313 articles marked ineligible were removed, and others were removed because their full texts could not be located. Of the 256 articles sought for retrieval, 206 were inaccessible. The researchers reviewed all 50 full-text articles of eligible abstracts, excluding 34 studies that either did not focus on community-based interventions supporting OVC, targeted populations other than OVC, or were conducted outside of Africa. Subsequently, 16 articles were reviewed to identify specific community-based interventions supporting OVC. Considering relevancy and compliance with the qualifying requirements, the remaining 16 articles were chosen, as indicated in Figure 1.

### Critical appraisal of included studies

The quality of the research was assessed using the Critical Appraisal Skills Programme (CASP) qualitative research checklist. The checklist, which aims to evaluate the merits and drawbacks of qualitative research methodologies, consists of ten questions that cover various methodological dimensions of a qualitative study. Designed as a teaching aid, the CASP checklist is particularly beneficial for individuals with minimal formal experience in evaluating qualitative research [12]. Given that the review is health-related, the tool is deemed appropriate for this context [12]. Ten studies were subject to the cohort study checklist, four went through the systematic review/literature review checklist and two went through the qualitative checklist. The majority of the included studies (*n =* 14) scored above 70%, and only two studies fell below 70% in the critical appraisal. The critical appraisals of the selected studies using the CASP checklist are summarised in Table 1.

**Table 1:** CASP appraisal outcome for selected studies.


AUTHOR, YEAR	STUDY DESIGN	CASP SCORE	PERCENTAGE

Gatsi, 2014	Mixed methods	7/10	70%

Mwoma et al., 2015	Mixed methods	9/10	90%

Breckenridge et al., 2019	Qualitative	7/10	70%

Ngwenya, 2015	Qualitative	8/10	80%

Nyakundi, 2017	Qualitative	6/10	60%

Martins, 2015	Qualitative	9/10	90%

Mbata, 2012	Qualitative	7/10	70%

Thwala, 2013	Mixed methods	7/10	70%

Mutenheri, 2011	Qualitative	8/10	80%

Chitiyo et al., 2010	Qualitative	8/10	80%

Nyawasha & Chipunza, 2019	Qualitative	8/10	80%

Adejimi et al., 2019	Qualitative	8**/**10	80%

Sitienei & Pillay, 2019	Qualitative	9/10	90%

Adelekan et al., 2015	Qualitative	6/10	60%

Omwa et al., 2011	Mixed methods	8/10	80%


### Data abstraction

Independently, the researchers extracted data and compared the data collected. Additionally, details on the study’s setting, design, objectives, target population, and published results were provided for all studies that meet the inclusion criteria. Using a data abstraction form created by the researchers for this review, one co-author performed the data abstraction. The reviewer revised the form multiple times during the data abstraction process to ensure that all fields were clear, relevant, and useful. For each study, the descriptive information on the population, sample size, demographic characteristics, declared research objectives, study design, and methods were abstracted. The main result of interest was any feature of a community-based intervention designed to assist OVC. Please refer to Table 2 for the characteristics of the included studies.

**Table 2:** Characteristics of included peer reviewed studies.


AUTHORS AND DESIGN	YEAR	OBJECTIVES	SETTING	PARTICIPANTS AND SAMPLE SIZE	STUDIES KEY CONCLUSIONS

1. Gatsi, mixed methods.	2014	*To get opinions from a group of Zimbabweans who have received psychological help*.	Bindura district, Zimbabwe	Teenage orphans(n = 60)	It was very evident that most support systems prioritize material needs such free food, clothes, and housing.

2. Mwoma et al., mixed methods.	2015	To comprehend the difficulties instructors, have in providing psychological support for OVC and the potential intervention techniques that may be used to ease these difficulties.	Soweto, South Africa	Educators (n = 42) and OVC in grade7 (n = 65)	Evidence-supporting findings show that just a little psychological support is given.

3.Breckenridge et al., qualitative research	2019	To address the impact of parental death/absence on orphaned children, with emphasis on those who experience parental death/absence due to HIV/ AIDS-related death.	Eastern Cape, South Africa	Orphans(n = 49)	In order to help the orphans’ caregivers, raise the children in a healthy and loving environment that will focus on reattachment difficulties , as well as to offer them access to specialists who can help them with any problems that may occur, NGOs must continue to work with them as the children mature and develop.

4.Ngwenya, qualitative research	2015	To investigate the difficulties faced by community-based programs in Mutare, Zimbabwe, for vulnerable children and orphans.	Mutare, Zimbabwe	Volunteers (n = 12)	The results also demonstrate that communities are devoted to the care and support of OVC by providing volunteer services, which the researcher has shown is true based on previous studies. Community-based treatments for OVCs confront problems, but the researcher has also validated this.

5.Nyakundi, qualitative research	2017	To investigate orphans’ and vulnerable children’s experiences receiving psychological help and their general wellbeing in Nairobi, Kenya.	Nairobi, Kenya	OVC and teachers (n = 57)	Findings again shown that OVC were vulnerable to a variety of risk situations not only at home owing to environmental and care variables, but also on their trip to and from school, as well as inside ECDE institutions.

6.Martins, qualitative research	2015	To investigate the participants’ experiences, beliefs, and issues with psychological assistance, as well as the perspectives of the responders	Khayelitsha, Western Cape	Foster mothers, social workers and administrator (n = 19)	The study’s findings are consistent with current literature that emphasizes the need of psychosocial care for orphaned and vulnerable children.

7.Mbata, Qualitative research	2012	To analyze the feasibility and preliminary efficacy of a psychological support group intervention for youth afflicted by HIV and their caregivers. To examine the effectiveness of the orphans and vulnerable children (OVC) program in schools.	Kwazulu- Natal, South Africa	OVC coordinators and OVC care givers (n = 13)	The study concludes that OVC programs in schools are not a cure-all for growth.

8.Thwala, mixed methods	2013	To investigate how culture affects the psychosocial growth of vulnerable children and orphans	Swaziland	OVC (n = 217) and their teachers (n = 87)	The children’s psychological needs are neglected in favor of the intervention’s emphasis on meeting their physical requirements.

9.Mutenheri, qualitative research	2011	To assess the James House program for vulnerable children and orphans	Cape town	OVC (n = 217)	The findings show that the James House program’s Isibindi model of care is a promising strategy for raising OVCs’ quality of life in Hout Bay’s informal settlement.

10. Chitiyo et al., qualitative research	2010	To determine if providing psychological support (PSS) to HIV-orphaned children has a positive impact on their academic performance.	Zimbabwe	Publications on orphans and vulnerable children made vulnerable by AIDS (n = 57)	Results indicate that during and after the intervention, the help given to these kids improved their performance in a number of areas, including education.

11. Nyawasha & Chipunza, ,qualitative research	2019	To evaluate the various psychological and empowerment programs given to orphans and disadvantaged kids in a Zimbabwean peri- urban area	Peri-Urban district, Zimbabwe	OVC(n = 30)	The findings demonstrate that there have been very few interventions created to address the psychological and empowerment needs of orphans and vulnerable children.

12. Adejimi et al. , qualitative research	2019	To highlight the accomplishments and effects of the World Bank-funded HIV Programme Development Project (HPDP) on providing treatment and assistance for OVC in Osun State, Nigeria	Osun State, Nigeria	OVC (n = 12353)	The FGD and KII reported that the project had a significant influence on changing OVCs’ lives.

13. Sitienei & Pillay, Qualitative research	2019	Examining the social, psychological, and educational support offered to orphans and vulnerable children (OVC) at a community-based organization (CBO) in Soweto, South Africa	Soweto, South Africa	OVC (n = 12)	The study’s findings demonstrate that CBO provided psycho educational and social treatments to OVC.

14. Visser et al. , mixed methods	2015	To report the assessment post-programme results of the ISIBINDI model, a community- based intervention to improve the physical and mental health of orphans and vulnerable children (OVC) in South Africa.	Soweto, South Africa	ISIBINDI ex- participants at 12 sites(n = 427) Nonparticipants (n = 177)	According to the evaluation, the ISIBINDI-model helps produce certain beneficial results that act as a barrier of protection for OVC. The review emphasized the significance of including those older than 18 in order to maintain decreased vulnerability.

15. Adelekan et al., qualitative research	2015	To outline the accomplishments of the OVC care and support program in Bayela State, Nigeria, as well as the implications for upcoming programming.	Bayelsta State, Nigeria	OVC(n = 5410)	The care, support, and protection of vulnerable children should not only concentrate on meeting their immediate needs for food, shelter, water, education, and clothing, but also on meeting their long-term developmental requirements that lessen their vulnerability, such as life skills, child protection, career training, food security, and household economic strengthening.

16. Omwa et al., mixed methods	2011	To demonstrate how the Northern Ugandan OVC group is under intense strain from a number of reasons.	North Central Uganda	Household heads fostering orphans(n = 20) Orphans(n = 20)	To address the difficulties of serving the demands of OVC, community activities are dynamic and continually changing through a variety of strategies.


### Ethical considerations

This review followed all ethical standards for research without direct contact with human or animal subjects.

## Analysis and synthesis

The researchers cleaned the data in the extraction sheet to ensure accuracy and consistency. To further ensure the quality of the studies, the characteristics of the included studies were presented, followed by a thematic analysis and a report of the findings. Since thematic analysis is commonly used to combine qualitative research findings, it was chosen for this evaluation. The results from the thematic synthesis are easily accessible, and the process is transparent. Thematic synthesis involves three levels of analysis, carried out in three stages. The information about the community-based interventions for OVC was first coded. A coding framework made up of codes drawn from the data was created. Similarities between codes were found during the second step of the examination. Codes were organised into descriptive themes to identify and characterise patterns in the data across studies, facilitating comparison of findings both within and between studies as part of the ongoing analytical process of constant comparison. Each theme was then entered as columns in a table, alongside the coded data from each study. Building analytic themes was a part of the third step of the thematic synthesis. This phase sought to go beyond the original presented data by using a study-by-study analysis and an interpretation of their significance in connection to the review research question. This comprises the analysis’s narrative component, which gives a narrative account of each topic.

Themes were developed from the selected articles included in this study, focusing on community-based interventions to support HIV and AIDS-affected OVC in Africa. The following themes emerged from the analysis: psychosocial support, education and skills training, protection and legal support, financial stability for OVC and their families, food and nutrition, and access to healthcare facilities. The identified themes and corresponding sub-themes are presented in Table 3.

**Table 3:** Key themes and sub-themes that emerged from thematic content analysis.


THEMES	SUB-THEMES

**1. Psychosocial support**	Memory box

	Lay counseling

	Child and adult support groups

	Leisure activities

	Debriefing sessions for caregivers

**2. Education and skills training**	Negotiating with school officials

	Supplies of school uniforms and stationary

	School work or after-school work

**3. Protection and legal support**	Secure shelter

	Advisory to SAPS

**4. Financial stability for OVC and their families**	Income generating projects training

	Savings and loan programs

**5. Food and nutrition support**	Food Deliveries

**6. Access to Health care facilities**	Referrals to the Department of Health

	Treatment compliance

	Collecting medication


## Psychosocial support

### Memory box therapy

According to the three studies out of sixteen studies included in this review, memory box is used by community-based organisations to offer psychosocial support to OVC [16,1]. Memory box therapy is one of the activity that encourages OVC to tell their story (narrative) and draw upon positive memories about the deceased loved one [13]. Memory box therapy, was reported to be one of the most soothing activities that help OVC in coping with loss and recovering more quickly [16,1]. These activities usually occur in OVC houses (on home visits), drop-in centres, resource centres, workshop locations or playgrounds.

### Lay counselling

Of the sixteen included studies, five report that personal counselling provided to OVC during home visits is the most common form of psychosocial support offered by community-based organisations [13,16,17,21,22]. Through frequent visits, caregivers and volunteers build strong relationships with OVC, allowing them to observe their emotional well-being and provide counselling when necessary. The counselling helps OVC cope with their circumstances in a more positive and resilient manner. Counselling as a form of psychosocial support helps the orphaned child to be able to share his/her worries and be helped to deal with stressful moment [13].

### Child and adult support groups

A support group refers to people with shared understandings or worries who provide each other with inspiration, relief, and advice [16,17,18]. The findings of this study show that CBOs are using support groups to offer psychological support to OVC.

### Leisure activities

The community-based organisations are giving OVC an opportunity to socialize with other children, which makes them very happy. Leisure activities made psychosocial support services available for children. The activities include a dancing competition, throwing the ball, dancing around the chair, a wheelbarrow race among others [22,23,24].

### Debriefing sessions for caregivers

The study findings reveal that the CBOs in Africa are providing counselling and debriefing to the orphans and vulnerable children as well as their families as a way of offering psychological support to OVC [18,19,20,22].

### Education and skills training

#### Negotiating with school official

Four of the included studies highlighted the importance of education for children’s physical, intellectual, emotional, and social development [13]. School attendance plays a crucial role in helping OVC to regain their sense of normality and to recover from the psychosocial effects of their experiences and disturbed well-being [13,14]. It has been reported that educational programs for OVC in African countries such as Burundi, Zambia, Lesotho, Kenya, and South Africa have shown that education interventions benefit individuals and entire nations, serving as a key driver of social and economic development [13,14,16,18]. In this regard, most of the articles in this study indicate that community-based organisations assist OVC by negotiating tuition exemptions with school officials. School exemption will also enable the OVC to access education in order to have a better future [13,14].

#### Supplies of school uniforms and stationery

According to four studies out of sixteen studies included in this review, many OVC in Africa have been able to resume school and complete basic education, something they could not have attained without the intervention of community-based organisations [24]. The community-based organisations support OVC’s education by providing them with school uniforms and stationeries such as pens, pencils, and textbooks [13,14,16,21]. The findings also revealed that among the OVC who received educational support, 44% received assistance with school fees, 61% with books, and 12.1% with school supplies [24].

#### School work or after-school care

Five of the studies analysed in this review reported that caregivers or volunteers in community-based organisations assisted OVC with their schoolwork or after-school care [13,14,16,21]. CBO staff and caregivers assist with registering OVC at schools and occasionally visit to monitor their progress. The findings revealed that the staff engaged with teachers to receive feedback on the children’s performance and attitude toward schoolwork. For child-headed families, caregivers provide ongoing support and monitor the completion of homework [13,16,18].

### Protection and legal support

#### Secure shelter

The findings of this review revealed that OVC were cared for within CBOs and were also provided with shelter [22]. Every child has the right to shelter, which provides protection from harsh conditions such as rain, extreme heat, violence, abuse, and environmental disturbances like noise [16,18,24]. Shelter is also important as an arena for privacy space, dignity and peace. In several African countries, including South Africa, Kenya, Uganda, and Botswana, numerous residential care institutions support OVC, achieving remarkable levels of success [22,24].

### Linking OVC with other departments

Although various grants are available for OVC in Africa, many OVC and their caregivers remain underprivileged and uneducated, limiting their ability to access these grants, which could significantly improve their education. Moreover, OVC and their legal guardians often face challenges in obtaining identification documents due to the lengthy and complex processes at the Department of Home Affairs, making independent access difficult [7,8,15]. The CBO facilitates access to the Department of Home Affairs for OVC, streamlining the process of obtaining identification documents, which are essential for accessing welfare benefits. In addition, many CBOs send personnel to the relevant government agencies to facilitate the processing of funds for OVC. Child support grants, foster care grants, and disability grants are among the key financial support available to OVC and their families. For many OVC, these grants serve as their primary source of livelihood [7,8,9,10,15,16].

#### Financial stability for OVC and their families

#### Income generating projects training

Some programs have focused on economically strengthening OVC and their families by empowering them through income-generating projects and providing ongoing support [13,16,18]. As a result, these OVC and their families are better able to support themselves and create a healthier environment for the children to thrive. OVC who struggled academically were given the opportunity to pursue livelihoods based on their talents. Community-based organisations trained OVC in various skills, including drama, art, music, hairdressing, and carpentry. Many OVC are now earning a living from the vocational skills they acquired through these programs [16,21].

#### Saving and loan programs

Education and emotional support programs alone cannot fully address the challenges faced by orphaned and vulnerable children, particularly those related to their ability to support themselves and ensure their overall well-being [16]. The findings of this review revealed that the CBOs implemented a new program designed to support orphaned and vulnerable children, as well as their caregivers, who are facing economic hardships. The review specifically found that community-based organisations (CBOs) utilised self-help groups to empower caregivers and promote their independence. CBOs encouraged caregivers to save money and lend it to each other. This encouraged them to work hard, to be independent and not rely on donations for survival [16,18,22]. Further, the findings indicated that CBOs encourage orphaned and vulnerable children to become self-sufficient and avoid relying solely on project funds [17]. As a result, caregivers and vulnerable children are better equipped to manage their businesses and generate a steady monthly income. Children who faced educational challenges were given the opportunity to use their natural talents to earn a livelihood. This is crucial because, without learning how to support themselves, these children and their families may become overly dependent and struggle to thrive. Furthermore, if the organisations supporting them were to discontinue their assistance, they would face significant difficulties in sustaining themselves [16,18,22,24].

#### Food and nutrition support

##### Food deliveries

Five out of the sixteen studies analyzed in this review indicated that CBOs in Africa provided food and nutritional assistance to OVC. The findings revealed that OVC gets varying food with good nutritional support levels, food deliveries, ready meals, food gardens, and animal gifts. The findings revealed that most CBOs regularly provide food and nutrition services, including gardening support and grocery delivery, within the same neighborhoods. Caregivers deliver food packages to resource centers while conducting home visits [11,13,14,16,18].

##### Access to healthcare facilities

#### Referrals to department of health

CBO staff refer caregivers to the Department of Health for medicines and vaccinations [6,8]. Some of the community-based interventions in Zimbabwe are facilitated by social workers through the Department of Social Welfare who issue, for example, medical treatment orders to OVC for them to receive free treatment at the government hospitals [13,15]. When caregivers visit a home and notice a child is unwell, they arrange for the child to see a doctor and receive free medical assistance [14,17]. If the children do not see a doctor, their illness may prevent them from attending school, rendering any money spent on school fees ineffective as they are unable to participate in their education.

#### Treatment compliance

Most CBOs in Africa offer general health services, primarily focused on assisting OVC in accessing health facilities when they are ill and lack a guardian. If a guardian is available, caregivers or volunteers remind them to vaccinate children [13,16]. They also support guardians by ensuring treatment adherence and, in some cases, collecting medication, particularly when OVC are under the care of their grandparents [6,8,13,15]. CBO staff also monitor OVC adherence to ARV therapy to ensure the effectiveness of their treatment [16].

#### Collection of medication

The findings of this review showed that CBO staff also monitored OVC adherence to ARV therapy to ensure the effectiveness of their treatment. Further, the findings revealed that CBO staff assisted OVC caregivers by reminding them of return dates to health facilities for vaccinations and medication collection [13,15,16].

## Discussion

In this review study, we examined and compared the approaches used by community organisations to support orphaned and vulnerable children in Africa. The review identified 16 studies examining various interventions across five countries. Comparing the interventions is challenging due to variations in the services offered by each program, the levels of support provided, and differing social contexts.

This systematic review identified six key types of community-based interventions supporting OVC in Africa including psychosocial support, education and skills training, economic empowerment for OVC and their families, protection and legal support, food and nutrition support, and general health care. Psychosocial support, including counselling services, is a crucial form of assistance for OVC. Counselling helps orphans navigate their circumstances with greater resilience and positivity [13].

Orphans and vulnerable children often suffer from considerable emotional trauma as a result of their respective family situations ranging from loss of one or both parents and the trauma that comes from living in various abusive circumstances. This, coupled with stigmatisation and marginalisation, leaves the OVC extremely vulnerable, in need of counselling and various other coping mechanisms [13,15,16,18]. Evidence from reviewed studies showed that psychosocial support gives OVC the skills, with which to cope with stress, trauma and other difficult situations that orphans and vulnerable children experience in life. Typically, programs to support vulnerable children focus on material needs, followed by education and skills training, but only some adequately handle the social and psychological needs of affected children.

The second community-based intervention identified to support OVC is education and skills training. Education and training are regarded as the most effective preventive measures to reduce the spread of infections. Through skills training, children learn many skills and good behaviours, such as avoiding early sexual activity and preventing HIV and AIDS and other sexually transmitted infections. They also learn how to prevent teenage pregnancy and use condoms when tempted to engage in sexual activity. Over the past decade, thousands of orphans and vulnerable children (OVC) in Indonesia have successfully completed their university education with the support of community-based organisations [2,18,22]. This is a direct impact of having the support they need to deal with emotional challenges brought about their situation.

The third form of community-based intervention supporting OVC in Africa identified in this review is the economic empowerment of both the children and their families. This review found that many CBOs operate in economically disadvantaged communities characterised by high unemployment rates. So, not only do some CBOs help OVC access social benefits, but they also offer sewing, beadwork, and chicken-raising training. The review also found that OVC were supported in starting small projects, such as vegetable gardens and selling sweets and popcorn, to generate income for their food and basic needs like soap [11,13,15]. Interventions that provide OVC with vocational training and keep them engaged within their communities are crucial for their long-term well-being and future prospects [15,16].

The fourth type of community-based intervention supporting OVC is protection and legal support, with CBOs fostering partnerships with other organisations to enhance their efforts. CBOs assist in reporting instances of child abuse or harm to the police and organisations dedicated to child protection. This helps ensure that the person who hurt the child is punished in court.

Food and nutritional support is another key form of assistance provided to OVC by community-based organisations, as identified in this review. Nutritional needs are essential, as proper nutrition contributes to improved health and overall well-being [6]. If OVC are not provided with adequate, nutritious food, their health will suffer, leading to poor concentration at school due to hunger. This can result in higher dropout rates and more children roaming the streets.

The sixth type of community-based intervention for OVC identified in this review is access to universal health care treatment. This intervention aimed at supporting OVC have also been effective in improving their health outcomes [16,18]. Health is approached through various means, including partnerships with both private and public hospitals, allowing OVC to access the free healthcare system [13,15,16]. As a result, there is a sense of protection, as diseases can be detected and treated early, allowing OVC to lead healthier lives.

## Implications to practice and support of OVC

Notwithstanding the recorded successes of community-based interventions supporting OVC, many CBOs face the challenge of insufficient funding to meet the needs of the ever-growing number of OVC [2,16]. The growing number of OVC now exceeds the limited financial resources. As a result, some of the interventions have been hit with reduced funding due to the global economic challenge. To guarantee these children a better life and future, many OVC have been incorporated into society through community-based organisations. Integration of all services is necessary to prevent program duplication. For example, when support is needed, medical, mental health, and nutritional services ought to be offered concurrently in the same neighbourhood, by the same organisation, and even to the same group of children. By eliminating stigma and referring to all children, including those who are HIV-positive, as “children-at-risk,” it became possible to effectively reach out to the HIV-positive population, a group that is often hard to access.

Furthermore, the children’s status was used as a requirement for selection. Community-based interventions should be assigned a better and more realistic aim to support more orphans and vulnerable children. For more detailed reporting, there should be a viable strategy and enough resources for the evaluation and monitoring procedure. A minimal set of preventive services should be offered when working with OVC, given their diverse needs. To address the needs identified in the household assessment, a situational analysis should be conducted, and strong connections should be established across the care system.

## Recommendations

The government should maintain and increase financial support for community-based organisations that assist OVC, aiming to strengthen services and enhance the outcomes of OVC programs. CBOs should constantly review their service delivery strategies and approaches to monitor whether their interventions are effective. Regular meetings should be held among OVC stakeholders, including the State Technical Working Group and a coalition of civil society representatives, to discuss key issues impacting OVC. These meetings should result in a communiqué and actionable plan to address OVC challenges at both provincial and national levels. Moreover, a resilience plan should be developed to safeguard the progress and success of community-based interventions, preventing setbacks in OVC support. The initiative can also reach additional OVC in the future thanks to its growth. Therefore, it is recommended that the goals and funding allocated to program stakeholders be thoroughly reviewed. There must be assistance from donors and implementers to facilitate the gathering of reliable empirical data. Several programs are promoted using an evidence-based strategy. To make this practicable, adequate monitoring and assessment are needed. As a result, it is necessary to create tools for collecting more effective data, examine existing ones, and re-train staff members on how to utilize the tools for effective reporting. Initiatives for OVC in the state should be community-owned and integrated with current initiatives.

## Limitations

The review had few limitations just like any other study. One of the limitations was that the review only reviewed articles from Africa and those written in English. Other studies published in different languages could impact the review. The review also considered articles from 2010 to 2021. Studies conducted before 2010 and after 2021 could impact the results. Despite these limitations, the information obtained was useful in shedding light on the type of support that the CBOs in Africa are providing to OVC.

## Conclusion

To sum up, our review found that community-based organisations, through their interventions, play a crucial role in improving the lives of OVC. Particularly, community-based organisations offer different forms of support to OVC. Community-based organisations also impact positively on the well-being of OVC. However, to be accessible and sustainable, community-based interventions need to be integrated into a wide social care system to ensure that their contribution in supporting OVC is formally recognised.
